# Microbial hydrogen oxidation potential in seasonally hypoxic Baltic Sea sediments

**DOI:** 10.3389/fmicb.2025.1565157

**Published:** 2025-04-04

**Authors:** Nicole Adam-Beyer, Christian Deusner, Mark Schmidt, Mirjam Perner

**Affiliations:** ^1^Geomicrobiology, Marine Geosystems, GEOMAR Helmholtz Centre for Ocean Research Kiel, Kiel, Germany; ^2^Benthic Biogeochemistry, Marine Geosystems, GEOMAR Helmholtz Centre for Ocean Research Kiel, Kiel, Germany

**Keywords:** hydrogen oxidation, hydrogen consumption, marine sediments, methane metabolism, methanogenesis, AOM, ANME, SRB

## Abstract

The majority of the organic matter (OM) degradation on the seafloor occurs in coastal regions. Since oxygen (O_2_) becomes quickly depleted in the top sediments, most of the OM decomposition is driven by microbial sulfate reduction (SR) and fermentation, the latter generating molecular hydrogen (H_2_). If the H_2_ is not consumed by hydrogenotrophic microorganisms and accumulates in the sedimentary porewaters, OM degradation is hindered. Despite the importance of H_2_ scavenging microorganisms for OM mineralization, the knowledge on H_2_ oxidizers and their constraints in coastal marine sediments is still quite limited. Here we investigated the role of H_2_ oxidizers in top (2 to 5 cm, suboxic-sulfidic) and bottom (18 to 22 cm, sulfidic) coastal sediments from a location exposed to seasonal hypoxia in the SW Baltic Sea. We used sediments from April, May and August, representative of different seasons. We spiked respective sediment slurries with H_2_ and incubated them for up to 4 weeks under O_2_-free conditions. H_2_ consumption potential, methane production and shifts in bacterial and archaeal 16S rRNA gene amplicons (generated from RNA) were assessed over time. The seasonal variations in sedimentary community compositions and pore water geochemistry already gave distinct starting conditions for the H_2_ enrichments. Sediments exposed to near anoxic bottom water conditions favored a microbial starter community exhibiting the highest H_2_ oxidation potential. Most of the observed H_2_ oxidation potential appeared associated with hydrogenotrophic sulfate reducers. The putative involvement of massively enriched ANME in H_2_ cycling in May 18 to 22 cm sediment horizons is conspicuous. While the differences in the observed H_2_ oxidation potentials in the studied sediment slurries are likely related to the (season-depending) overall redox state of the sediments and interstitial waters, the influence of microbial interconnections could not be fully resolved and evaluated, demonstrating the need for further consumption- and community-based studies.

## Introduction

With 70% of our planet’s surface being covered by marine sediments, they represent the largest reservoir of organic carbon on Earth and play a pivotal role for global carbon cycling (Middelburg et al., 1993). Although coastal sediments cover a relatively small area of the seafloor (9%), they account for most of the organic carbon decomposition (87%) (Middelburg et al., 1993). Most of the organic matter (OM) degradation in organic-rich marine sediments is driven by fermentation and microbial sulfate reduction (SR), as oxygen (O_2_) becomes quickly depleted in the top sediments (Jørgensen, 1982).

Fermentation of OM is the central pathway of generating molecular hydrogen (H_2_) in sediments (Laanbroek and Veldkamp, 1982; Dolfing, 1988). Accumulation of H_2_ already in the nM-range renders fermentation thermodynamically unfeasible; thus, fermentative OM degradation becomes limited or even stops if H_2_ is not scavenged rapidly by hydrogenotrophic microorganisms (Wolin, 1976; Hallenbeck, 2009). The control of *in situ* H_2_ concentrations occurs through syntrophic interactions between hydrogenogenic and hydrogenotrophic microbial processes, which generally rely on short-range interactions between individual microbial organisms in close spatial proximity to each other. In accordance with the thermodynamic constraints of syntrophic metabolisms and interactions, H_2_ turnover is fast and its residence time is very short. Consequently, measured H_2_ concentrations in sediment porewaters are commonly quite low (typically <60 nM) (Novelli et al., 1987; Novelli et al., 1988; Hoehler et al., 1994, 1998; Lin et al., 2012). Due to the broad spectrum of hydrogenotrophic microorganisms in O_2_-free sediments, including nitrate-, manganese-, sulfate-, and iron-reducers as well as methanogens, a huge variety and variability of syntrophic interactions exists (Lovley and Goodwin, 1988; Hoehler et al., 1998; Vandieken et al., 2014).

Consequently, phylogenetically and functionally diverse microbes are capable of utilizing H_2_ as energy source (Greening et al., 2016; Adam and Perner, 2018a; Lappan et al., 2023) and an even larger enzymatic repertoire is likely masked among the uncultured microbial majority (Adam and Perner, 2018b). In anoxic sediments, common H_2_ utilizers include sulfate reducing Bacteria (SRB), producing toxic hydrogen sulfide, hydrogenotrophic methanogens, generating the greenhouse gas methane, and homoacetogens producing acetate (Lovley et al., 1982; Hoehler et al., 1999; Ragsdale and Pierce, 2008). Given that SR is thermodynamically more favorable than both hydrogenotrophic methanogenesis and homoacetogenesis, SRB have been documented to outcompete hydrogenotrophic methanogens and, respectively, homoacetogens when H_2_ levels fall below a particular threshold concentration (Oremland and Polcin, 1982; Lin et al., 2012). In contrast, at high H_2_ concentrations, as it is often imposed in physiological experiments, simultaneous occurrence of several H_2_-scavenging processes is possible (Holmer and Kristensen, 1994; Lay et al., 1998; Timmers et al., 2015). Conclusively, the competition for H_2_ determines the flow of carbon and electrons and the degree at which hydrogen sulfide, methane and/or acetate is generated, impacting the sediment ecosystem and beyond. Despite the profound role that H_2_ has for OM break down and the type of generated products, the knowledge on the ecophysiology of H_2_ converting microbes in these sediments is still quite limited.

So far only a limited number of studies have attempted to disentangle the H_2_ oxidation potential, associated microbial communities and dynamic response to changing environmental conditions in marine anoxic sediments (Hamann et al., 2016; Dyksma et al., 2018). This is true in particular for coastal sediments, which are usually exposed to seasonal variations with respect to bottom water temperature and chemistry. Given environmental dynamics, steady-state conditions - assumed to underly syntrophic interactions in anoxic sediments can most likely not be established, thus a stable H_2_-metabolic community is not to be expected. The Boknis Eck long-term study site, in the SW Baltic Sea is an ideal natural laboratory to test seasonal dynamics in coastal marine sediments. It is located at 28 m water depth, sediments are rich in OM (~5% wt. %) (Dale et al., 2013; Wallmann et al., 2022) and are exposed to late summer seasonal hypoxia (O_2_ < 63 μM) /anoxia (no O_2_) (Dale et al., 2013; Perner et al., 2022). Peak sulfate reduction rates (SRR) in summer are considerably higher and at the surface as opposed to SRR in winter and early spring where they are 10-fold lower and maxima are reached below the first centimeters (Dale et al., 2013; Perner et al., 2022). Experiments targeting methanogenic processes in these sediments have suggested that SRB and methanogens are both active in the sulfate reducing zone, but that methanogens are using H_2_ alternative substrates such as methylated compounds or methanol (Maltby et al., 2018). Anaerobic oxidation of methane (AOM) and anaerobic methane oxidizing Archaea (ANME) have been documented down to depths of 25 cm at Boknis Eck (Treude et al., 2005; Perner et al., 2022).

In this study, we aimed to elucidate the H_2_-oxidizing potential and microbial growth in the presence of sub-millimolar H_2_ concentrations. For that, we designed an experimental setup where we offered H_2_ in excess (2% in headspace or ~ 1.2 μM in solution) to marine sediments and kept them under anoxic conditions. Coastal sediments at Boknis Eck (SW Baltic Sea) exposed to seasonal hypoxia were used for incubation experiments. Sediments were collected in April where bottom waters were fully oxic, in May where bottom waters started to become O_2_ depleted and in August, where bottom waters were near anoxia. H_2_ consumption rates and microbial shifts in the sediment slurries were monitored under anoxic conditions for up to 4 weeks to address the full H_2_ oxidizing potential when H_2_ is available in excess relative to *in situ* conditions.

## Materials and methods

### Sample collection

**Figure 1:**
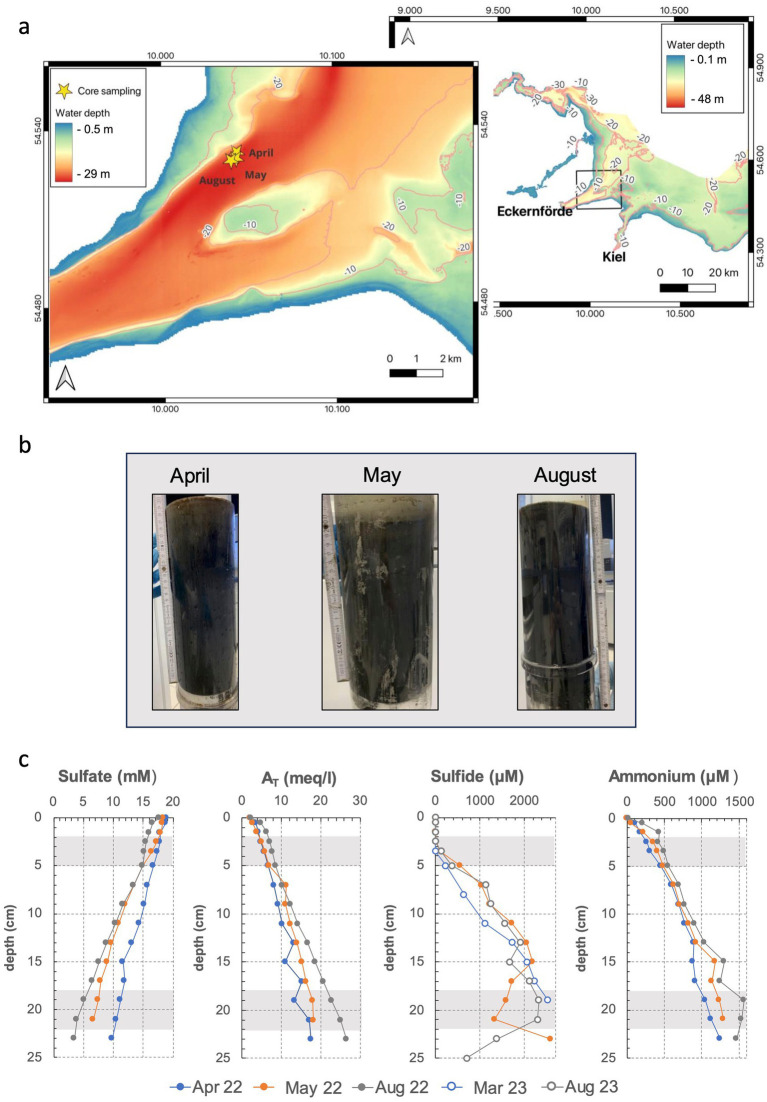
Map of the sampling area in the South-Western Baltic Sea **(a)**, sampled sediment cores **(b)** and porewater profiles of sulfate, total alkalinity (A_T_), sulfide and ammonium **(c)**. Geochemical data is derived from (Dale et al., 2024), except for May 2022 and August 2023 (M. Perner, unpublished data). For April and August 2022, no sulfide data are available.

Sediment samples were collected in April, May and August 2022 in close proximity to the time series station Boknis Eck in the Eckernförde Bay (South-West Baltic Sea, Germany; see Figure 1a) (54°31.76’ N, 10°02.50′ E). Sediment cores (Figure 1b) were taken using a Minicorer (MIC) device, transported to land within 3 h of sample retrieval and kept at 4°C until the subsampling for setting up hydrogen incubation experiments was performed (max. 24 h after core retrieval). Pore water extractions were performed from neighboring sediment cores at the respective sampling days (profiles of selected biogeochemical parameters can be found in Figure 1c).

### Hydrogen incubation experiments

For hydrogen consumption measurements sediment cores were subsampled in 2–5 cm and 18–22 cm horizons and mixed to obtain a homogenous dispersion of the sediment communities in each subsample. For later analyses of the microbial communities, aliquots of the subsamples were immediately frozen at −70°C. Under anaerobic conditions (5:95 H_2_:N_2_ atmosphere) aliquots of 1 mL of the subsampled sediments were distributed into 60 mL serum bottles filled with 10 mL of a modified, organic-free artificial seawater (MJ) medium (Sako et al., 1996; Hansen and Perner, 2015). Negative controls were set up by adding formaldehyde to a final concentration of 4%. Each sample and control slurry was set up in triplicates and purged with a gas mixture (2:20:78 H_2_:CO_2_:He) to obtain a H_2_ concentration of 2% in the head space. H_2_ consumption was monitored by weekly measurements of the H_2_ concentrations in the head space as previously described (Hansen and Perner, 2015; Adam et al., 2021), over a time period of up to 26 days. All measurements were performed using a TRACE GC Ultra gas chromatograph (ThermoFisherScientific, Waltham, MA, United States), equipped with a ShinCarbon ST 100/120 column (Restek Corporation, Bellefonte, PA, United States) and a Pulsed Discharge Detector (Vici Valco Instruments, Houston, TX, United States) with a detection limit of 10 ppb. Head space samples of the sediment incubations were diluted 1:124 for measuring in the calibrated range of up to 191 ppm H_2_ (RMSE 1.46 ppm). The methane concentration in the head space was calculated from the same chromatograms used for H_2_ measurements but using a separate calibration (RMSE 20.11 ppm in the range of 597–1792 ppm) and manual integration of the methane peak. During the incubation period, all samples and controls were kept at the respective *in situ* bottom water temperature measured at the date of the sediment core retrieval: 4°C for April, 5°C for May and 10.5°C for August incubations.

At the beginning and termination of the incubation experiments 1 mL subsamples of each sediment slurry (incl. controls) were taken and fixed with formaldehyde (4% end concentration) at 4°C overnight. After harvesting (5 min, 5,000 g, 4°C) and two wash steps with 1 mL of 1x Phosphate Buffered Saline solution (PBS), the fixed cells were stored in 1 mL of PBS:Ethanol (1:1) at −20°C. Subsequent DAPI-based cell enumerations were performed as described in (Perner et al., 2022) and used to calculate hydrogen consumption rates as previously described (Hansen and Perner, 2015). For taxonomic analyses of the respective microbial communities at the end of the incubations, sediment slurries (excl. controls) were harvested at 20,000 g (4°C) for 30 min, washed with 10 mL of 1xPBS and frozen at −70°C.

### Nucleotide extractions, 16S rRNA and mcr gene analyses

RNA and DNA extractions of the original sediment subsamples (t0) and the harvested slurries were performed using the NucleoBond RNA Soil Mini Kit with the additional DNA set according to the manufacturer’s instructions (Macherey-Nagel, Düren, Germany). Residual DNA was removed from the extracted RNA by means of the RapidOut DNA Removal Kit (ThermoFisher Scientific) and cDNA synthesized with the SuperScript™ VILO™ cDNA synthesis kit (ThermoFisher Scientific) following the manufacturer’s protocols. Purification of the cDNA was performed using the clean and concentrator-5 cleanup kit (Zymo Research, Irvine, CA, United States) according to the cDNA protocol. The V3-V4 regions of bacterial 16S rRNA genes and V4-V5 regions of archaeal 16S rRNA genes were amplified from the purified cDNA and prepared for Illumina MiSeq sequencing using the Bact_341F/805R, Arch_519F/915R and Arch 524F/958R primer pairs as previously described (Adam et al., 2019). Equimolar pools of purified amplicons were sequenced in a 2×300 paired-end sequencing run on Illumina’s Mi-Seq platform.

Demultiplexed raw sequences were processed in the Qiime2 environment (Bolyen et al., 2019) following a previously reported workflow (Adam-Beyer et al., 2023). Prior to merging with the dada2-plugin (Callahan et al., 2016), primer sequences were removed and the individual raw sequences were trimmed to 260 nucleotides, each. A pretrained classifier based on SILVA database release 138 (Quast et al., 2013) and the feature-classifier plugin (Bokulich et al., 2018) were used for taxonomic assignments. After removal of contaminating sequences (i.e., eukaryotic and chloroplast sequences as well as bacterial sequences in the archaeal data set and vice versa), phylogenetic trees were calculated using the “align-to-tree-mafft-fasttree” pipeline (Price et al., 2010). Relative abundance calculations, generation of tax plots and differential abundance analyses were performed using the microeco package (Liu et al., 2021) in R (version 4.3.1; https://www.r-project.org/). For differential abundance calculations, the random forest and differential test approach was applied after a rarefication to 9,500 sequences/sample for bacterial and 7,000 sequences/sample for archaeal analyses.

Due to the conspicuously high enrichments of ANME 16S tags present only in the 18 to 22 cm sediment incubations from May and their ambiguous involvement in methane cycling in the corresponding samples, genes encoding for the methyl coenzyme M reductases (Mcr, key enzyme for methanogenesis and methanotrophy) of methanogens, ANME-1 and ANME-2 were PCR-amplified from DNA extractions of the May 18–22 cm samples. Primer pairs used for the amplification were mcrI (Springer et al., 1995) for *mcr* genes of hydrogenotrophic methanogens, ANME-1-mcrI for ANME-1 related *mcr* genes and mcrANME-2 for ANME-2 related *mcr* genes (Lever and Teske, 2015). Amplification was performed using the Phusion High-Fidelity PCR-Kit (Thermo Scientific) with the following conditions: initial denaturation for 30 s (98°C), followed by 31 cycles of denaturation (10 s at 98°C), annealing for 15 s (46°C for mcrI, 57°C for ANME-1-mcrI and mcr-ANME-2) and extension (15 s at 72°C). Final extension was carried out for (5 min at 72°C). PCR products were checked for the correct length via agarose gel electrophoresis: 470 bp for mcrI, 480 bp for ANME-1-mcrI and 155 bp for mcr-ANME-2. After purification with the clean and concentrator-5 purification kit (Zymo Research), PCR products were cloned into *E. coli* DH5 *α* cells using the pGEM-T vector system (Promega, Madison, WI, United States) according to the manufacturer’s instructions. Colony-PCRs of 20 white colonies each were performed for ANME-1-mcrI and mcr-ANME-2 clones using common M13 primers and the Phusion High-Fidelity PCR Kit as detailed above, with the exception of an annealing temperature of 58°C. Purified PCR products were sent for Sanger sequencing with the M13 forward primer at Eurofins Genomics (Cologne, Germany). Vector contaminations were removed and the mcr-sequences were aligned with reference mcr sequences using Bioedit (Hall, 1999). A phylogenetic tree was constructed with the MEGA X software using the maximum-likelihood method with a Tamura-Nei model and bootstrapping with 1,000 replicates (Kumar et al., 2018).

### CARD-FISH and cell counts

For all sediment slurries cell numbers were determined by DAPI staining as described above. In order to monitor if the increase in ANME-related 16S rRNA gene tags coincides with an overall increase of archaeal cell abundances, for the 18 to 22 cm sediments from May CARD-FISH (catalyzed reporter deposition-fluorescence *in situ* hybridization) was additionally performed using the EUB I and EUB II as well as the Arch915 probes for Bacteria (EUB I and II) and Archaea (Arch915), respectively (Pernthaler et al., 2002). Preparation of filters, permeabilization and hybridization were performed as previously described for sediment samples from Boknis Eck (Perner et al., 2022). Relative abundances of bacterial and archaeal cells were determined by comparing enumerations of hybridized cells against DAPI-stained cells.

### Data availability

Raw reads of 16S rRNA gene amplicons and *mcr* genes were deposited at the National Center for Biotechnology Information (NCBI) and can be accessed under BioProject PRJNA1213115.

## Results

In order to assess the H_2_ consumption potential and shifts in H_2_-metabolizing organisms in seasonally hypoxic sediments from the SW Baltic Sea, we monitored microbial H_2_ utilization and methane cycling in H_2_-spiked sediment slurries. Samples were taken from 2 sediment horizons (near top and bottom of MIC cores, i.e., 2–5 cm and 18–22 cm, respectively) of three different months (April, May and August), reflecting the seasonality. Additionally, cell numbers were determined and RNA extracted to generate 16S rRNA genes aiming to link H_2_ consumption with community shifts in the incubation experiments. For the May 18 to 22 cm sediment slurries, the presence and diversity of *mcr* genes (encoding for methyl coenzyme M reductase) were additionally determined based on DNA, as well as quantitative shifts of Bacteria relative to Archaea documented via CARD-FISH. These analyses were limited to the May bottom slurries, as only these exhibited a massive enrichment in ANME-related 16S rRNA gene tags.

### Hydrogen consumption rates and monitored methane concentrations in sediment slurries

#### Hydrogen consumption

**Figure 2:**
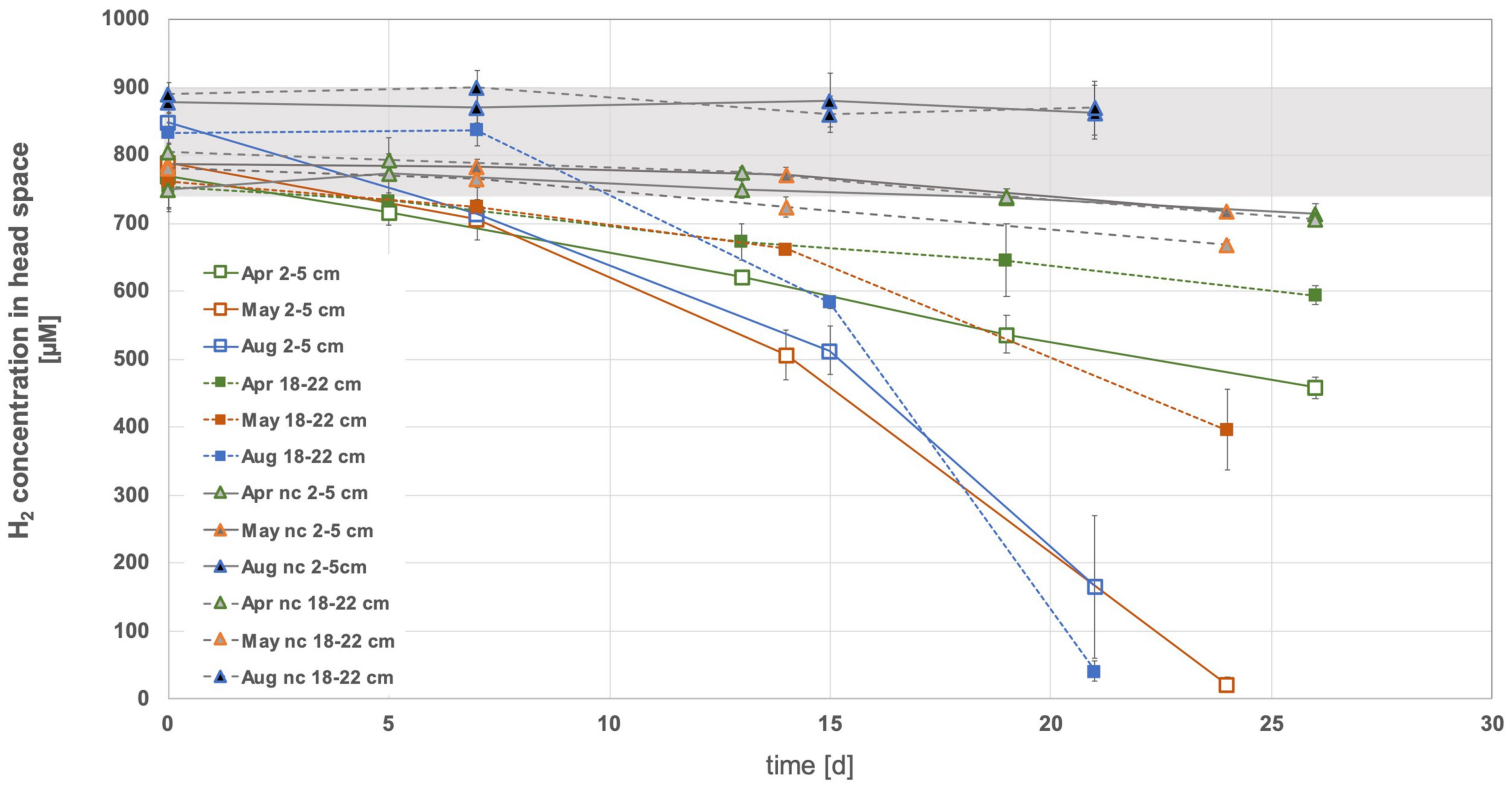
Hydrogen consumption in the headspace of sediments slurries with top (2 to 5 cm) and bottom (18 to 22 cm) sediments from April, May and August 2022. Sample names where *nc* precedes the month denote the negative controls. These were sediments treated with formaldehyde and where it was assumed that microbial activity ceased. The grey area denotes the range of hydrogen at the start of the experiment.

**Figure 3:**
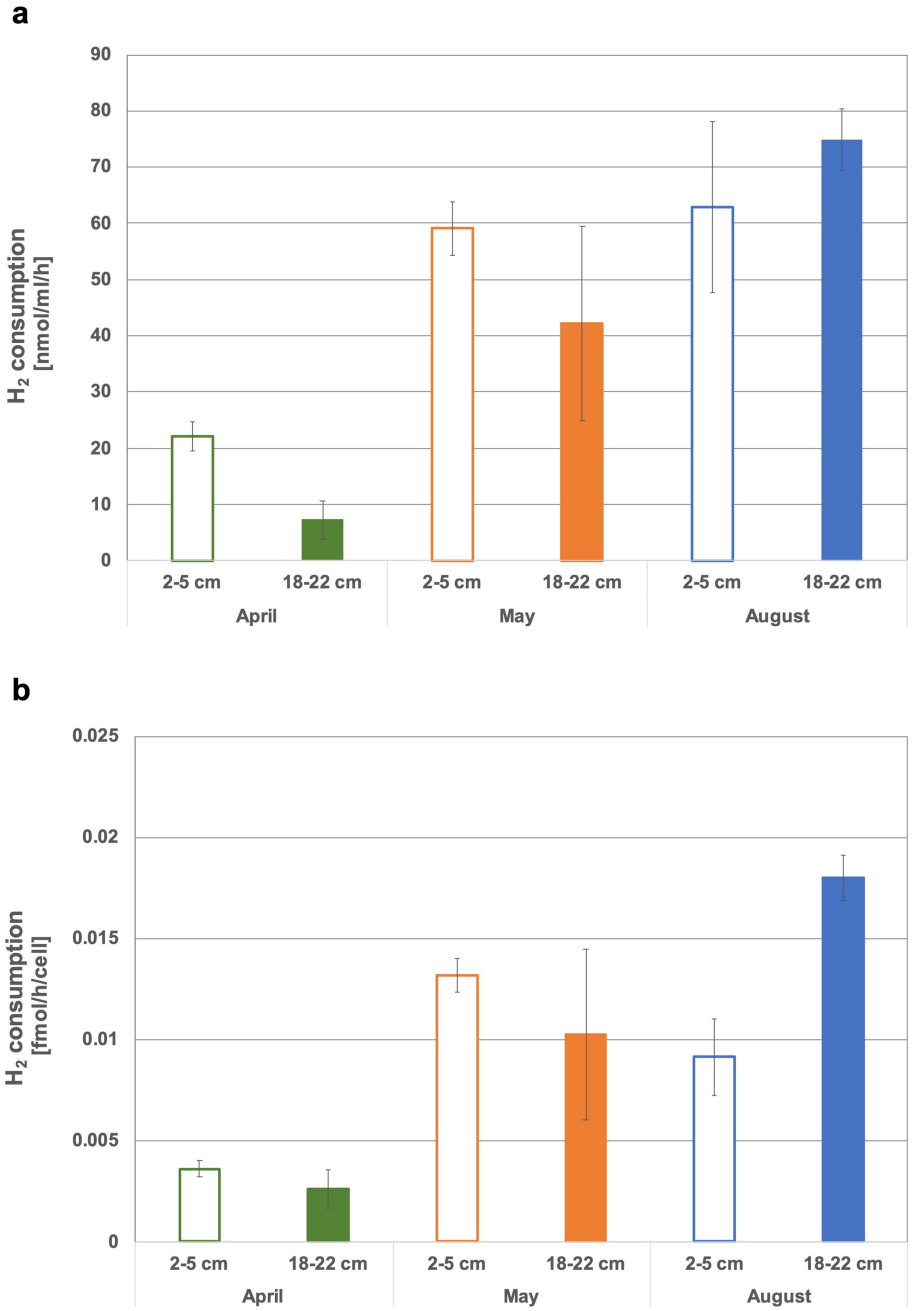
Hydrogen consumption of incubated sediment slurries per volume **(a)** and per cell **(b)**.

In the head space of the sediment slurries using top sediments (2 to 5 cm) from May and August and bottom sediments (18 to 22 cm) from August, on average nearly all of the offered H_2_ (i.e., 35–40 μmol equivalent to ~2% in headspace) was consumed within 3 weeks (Figure 2, Supplementary Figure S1). In contrast, even after approx. Four weeks of incubation, more than half of the initially added H_2_ was still present in both tested April sediment horizons and in the bottom May sediment slurries (exception was sediment slurry II of bottom sediments from May, where all H_2_ was used) (Supplementary Figure S1). In the April sediment slurries H_2_ consumption dropped at a relatively constant rate over time. The May and August incubations appeared to experience a H_2_ consumption boost after about one to 2 weeks (Figure 2), suggesting that microbial communities had to adapt, microbial growth had to overcome a lag phase or specific metabolic byproducts had to be produced before elevated H_2_ consumption could kick in. In April and May slurries, more H_2_ was consumed in the top than in the bottom sediments (Supplementary Figure S1). The April slurries exhibited the by far lowest consumption rates (7.3 ± 3.4 and 22.1 ± 2.7 nmol H_2_ mL^−1^ h^−1^ sediment ^−1^ or 0.002 ± 0.0009 and 0.003 ± 0.0004 fmol H_2_ cell^−1^ h^−1^, respectively, the latter assuming that all cells are using H_2_). The highest rates measured in bottom August sediments were up to 10 times higher than the lowest rates for April sediments (up to 74.8 ± 5.5 nmol H_2_h^−1^mL sediment ^−1^ or 0.018 ± 0.001 fmol H_2_ cell^−1^ h^−1^) (Figures 3a,b). When considering H_2_ consumption per cell, rates in May and August top and May bottom sediments were comparable, whereas those from August bottom sediments were 1.4 times higher (Figure 3b). Overall, the H_2_ consumption rates per volume and hour were in the upper range of and/or up to 2.7-fold higher relative to those determined for hydrothermal fluid samples in previous H_2_ supplemented fluid incubations (Perner et al., 2011; Perner et al., 2013). In stark contrast, the per cell estimates of H_2_ consumption was up to 17,000-fold lower than rates observed in previous H_2_ incubation experiments of hydrothermal fluids (Perner et al., 2013). Consequently, either only a small proportion of the microbial community is in fact consuming H_2_ or many cells may have the H_2_ consumption ability but are doing this at extremely slow rates.

#### Monitored methane contents

**Figure 4:**
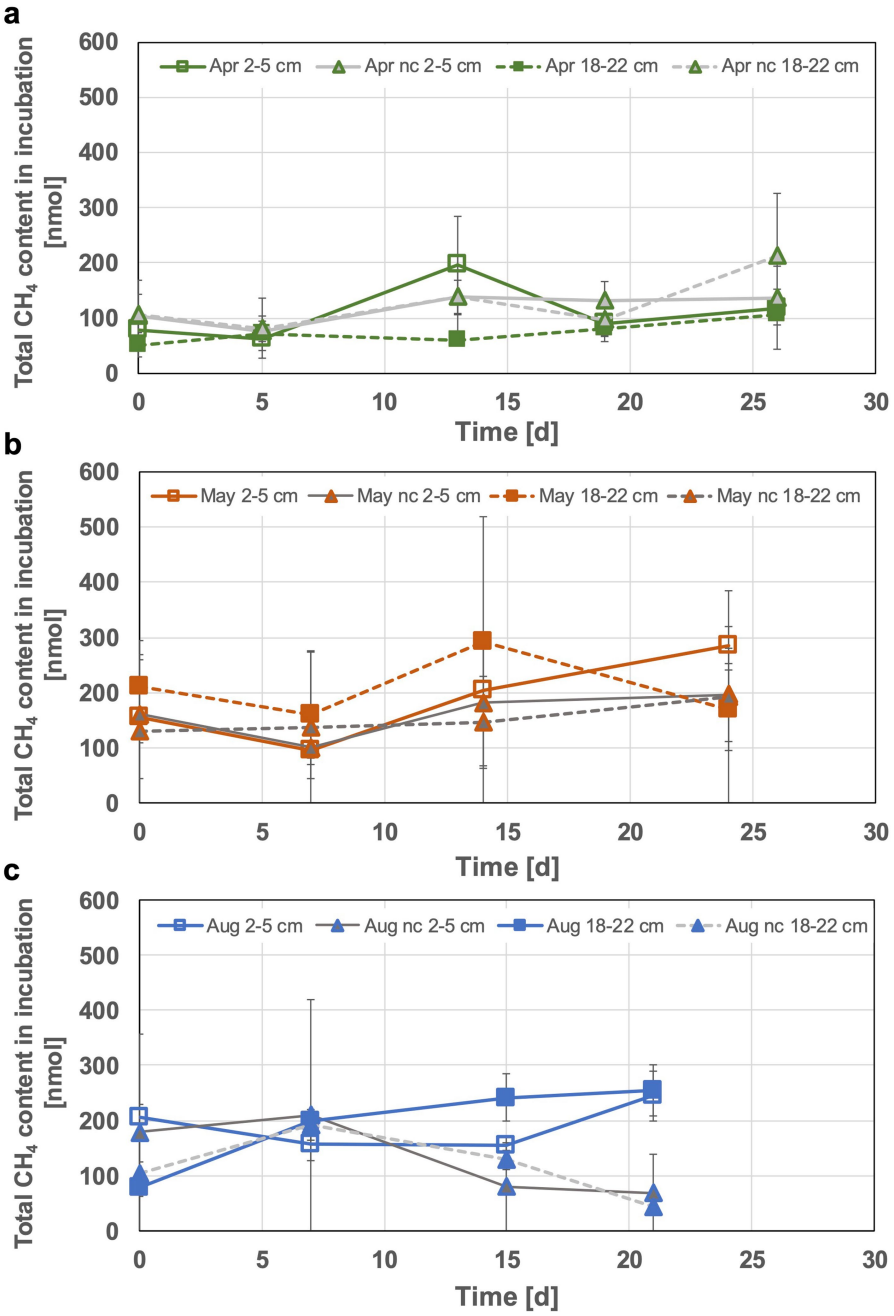
Monitored total methane content in the sediment incubations of April **(a)**, May **(b)** and August **(c)**. The 2–5 cm incubations are displayed with solid lines, 18–22 cm incubations with dashed lines and nc denotes formaldehyde treated incubations (negative controls).

Methane increase in head spaces was visible, albeit generally low and highest methane accumulation of up to 0.3 μmol (in May bottom incubations) was still a factor of 100 lower compared to total H_2_ consumption. However, especially during the second week of incubation the increase of methane concentrations indicated active methanogenesis in some of the incubated sediment slurries. The 2–5 cm incubations in April for example, showed an up to 3-fold increase in methane content (57% more than in the respective negative control, see Figure 4). The produced methane in April top sediment incubations appeared to be consumed during the third week, leveling off at the initial methane content. In case of the May incubations a slight initial methane consumption was observed for both of the incubated sediment depths, followed by an apparent methane production phase. This lasted until the end of the experiment for the 2 to 5 cm incubations, while it was followed by a decrease of the methane content in the 18 to 22 cm incubation during week three. Overall, the methane contents in the May incubations were higher (up to 50% during the peaks in the second week) than those observed during the other months (Figure 4). Interestingly, the 18–22 cm incubations of August sediments were the only ones characterized by a constant increase in methane content. The top sediment incubations from August were characterized by starting methane contents and an initial decrease similar to the bottom incubations from May. The methane consumption phase however, only started in incubation week three. The final methane contents were nearly the same for both sediment layers of the August incubations (Figure 4). The observed methane accumulation and consumption indicate the fine tuning among the communities for cycling biologically generated methane or specific shifts in methane-metabolizing communities at increased H_2_ concentrations.

### Microbial community shifts in sediment slurries

#### Community compositions at the start of the experiments

**Figure 5:**
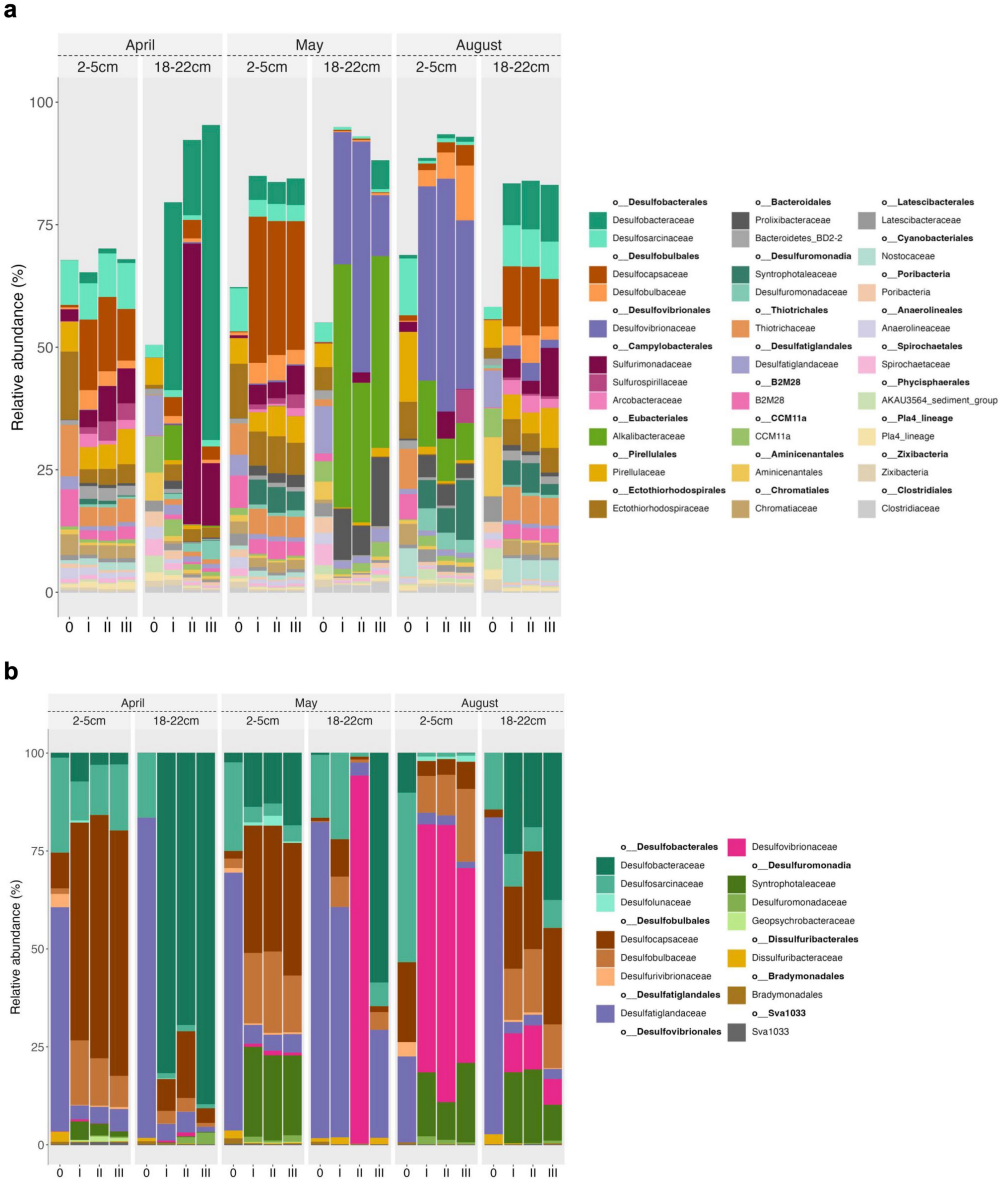
Taxonomy plots based on RNA of 16S amplicons on a family level for the 30 most abundant bacterial taxa **(a)** and putative Sulfate Reducing Bacteria (SRB) **(b)**. The numbers at the bottom of the graph denote the respective communities at the start of the experiments (0) and at the end of the experiment in triplicates (I, II, III). The SRB subplot **(b)** is scaled to 100%.

The most abundant 30 taxa made up 62 to 69% of the bacterial 16S rRNA gene tags of the top (2 to 5 cm) in April, May and August consisting of SRB, namely *Desulfosarcinaceae* (mainly Sva0081) and *Desulfatiglandaceae*, sulfur oxidizing Bacteria (SOB) affiliated with *Ectothiorhodospiraceae*, *Chromatiaceae* and uncultured *Thiotrichaceae*, *Sulfurimonadaceae*, B2M28, and organotrophic *Pirellulaceae* (Figures 5a,b). The high proportions of less abundant taxa (>30%) further suggest a high phylogenetic diversity in the sampled top sediments. In the August top sediments organotrophic *Pirellulaceae* and among the SRB *Desulfocapsaceae* were at least twice as abundant, and *Ectothiorhodospiraceae* and *Desulfatiglandaceae* were roughly half as abundant as in the April and May starter sediments (Figure 5). The initial microbial communities of the slurries reflected the conditions in the natural sediment horizons observed in former years (Perner et al., 2022).

**Figure 6:**
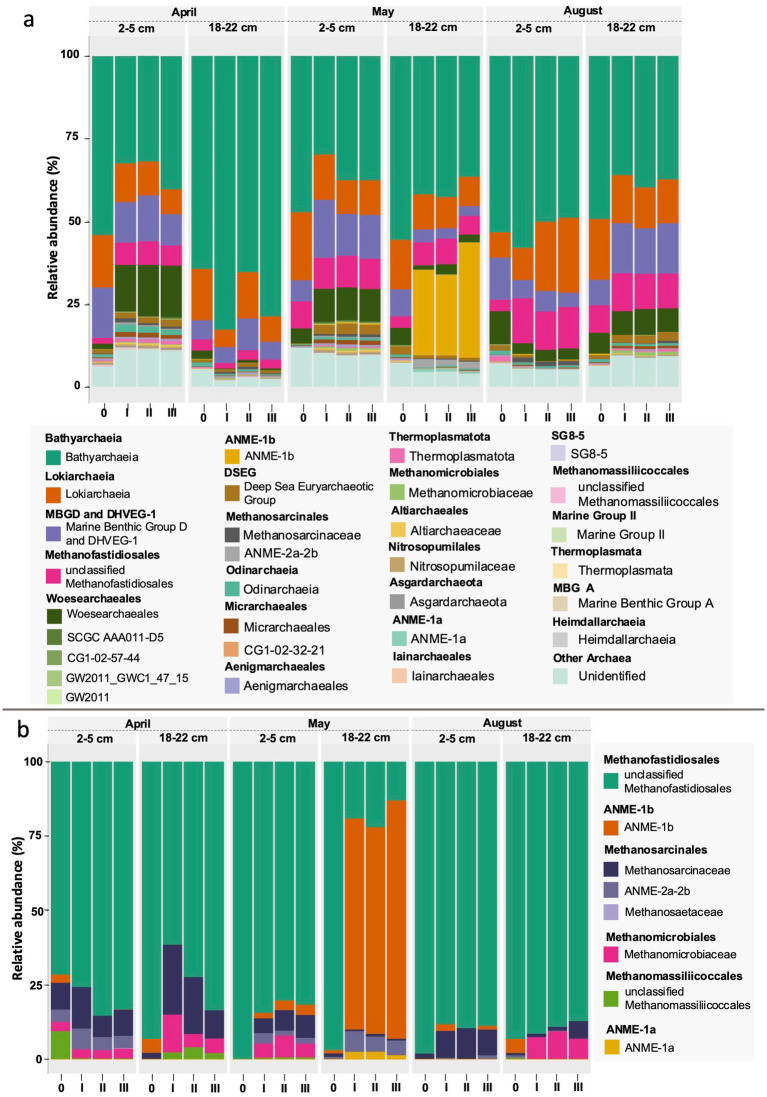
Taxonomy plots based on RNA of 16S amplicons on a family level for the 30 most abundant archaeal taxa **(a)** and traditional methanogens and anaerobic methane oxidizers (ANME) **(b)**. The numbers at the bottom of the graph denote the respective communities at the start of the experiments (0) and at the end of the experiment in triplicates (I, II, III). The methanogens/ANME subplot **(b)** is scaled to 100%.

The bottom sediments (18 to 22 cm) were characterized by a higher proportion of less abundant taxa (42 to 50%). Among the most abundant 30 taxa were mainly *Desulfatiglandaceae* and less *Desulfosarcinaceae*, few SOB mainly of *Ectothiorhodospiraceae* (< 1%), and organotrophic *Pirellulaceae*, CCM11a, *Aminicenentales*, and *Latescibacteraceae*. Again, this largely compares to what has been described for Boknis Eck sediment cores before (Perner et al., 2022). As already noted during previous sampling campaigns at Boknis Eck, the archaeal community was similar in all cores and remained remarkably constant during the seasons and the two depth horizons (Perner et al., 2022). Archaea were mostly (78–89% of archaeal 16S tags) dominated by members of the Bathyarchaeia, Lokiarchaeia, Marine Benthic Group D, *Woesearchaeales* and Deep Sea Euryarchaeotic Group (DSEG) (Figure 6a). Albeit in predominantly small proportions (<10%), ANME and traditional methanogens (e.g., unclassified *Methanofastidiosales* and *Methanosarcinaceae*) were present in all starter communities except for May and August top sediments (lacking ANME) (Figure 6).

#### Community shifts after 3 weeks of incubations

During the course of the experiment the microbial communities changed considerably in the sediment incubations (Figures 5, 6, Supplementary Figure S2). Depending on the sediment slurry, the bacterial communities exhibited strong enrichments mostly pronounced in potential SRB, some *Gammaproteobacteria* typically characterized as SOB, and *Alkalibacter* (Firmicutes) (Figure 5, Supplementary Figure S2). Interestingly, among the Archaea, the most pronounced shift was an increase in certain groups of ANME in the bottom sediments of May slurries (Figure 6).

*Top sediments.* During the experiments the top sediment slurries of April and May became enriched in members of the *Desulfobacterium catecholicum* group and *Desulfotalea* genus (*Desulfocapsaceae*) (particularly in the May sediments), and in *Sulfurimonadaceae* but depleted in *Thiogranum* spp. (*Ectothiorhodospiraceae*) (less so in May), *Desulfatiglandaceae*, and B2M28 (uncultured *Gammaproteobacteria*). Furthermore, *Syntrophotaleaceae* notably increased (up to 5%) during May top sediment incubations. *Desulfosarcinaceae* were unchanged for April but depleted in May. August top sediments became enriched in *Desulfovibrionaceae* (from <0.1% to up to 47%), *Desulfobulbaceae* (mainly of the *Desulfobulbus* genus), *Alkalibacteraceae* and *Syntrophotaleaceae* during the course of the experiment (Figure 5). In all sediment incubations the archaeal communities were generally dominated by Bathyarchaeia, regardless of the month or depth horizon (Figure 6). In April and May top sediment incubations *Woesearchaeales* became significantly enriched (10-fold and 2-fold, respectively) but their abundance decreased approximately 3-fold in August top sediments relative to the starter top sediment community (Figure 6a). Relative to the beginning of the sedimentary archaeal communities, abundances of members of the Marine Benthic Group D were slightly reduced in April, more than doubled in May, and were roughly halved in August top sediments over time. Lokiarchaeotal 16S rRNA gene amplicons were predominantly inconsistent in trends for the different slurries, while the abundances of unclassified methanogenic *Methanofastidiosales* increased during all incubations of the 2 to 5 cm sediment horizons (Figures 6a,b). This effect was differently pronounced for the distinct top sediment incubations: while the increase was approximately 1.5-fold in May, in August incubations it was roughly 3.6-fold and peaked with approximately 4-fold higher abundances of *Methanofastidiosales* at the end of the April incubations.

**Figure 7:**
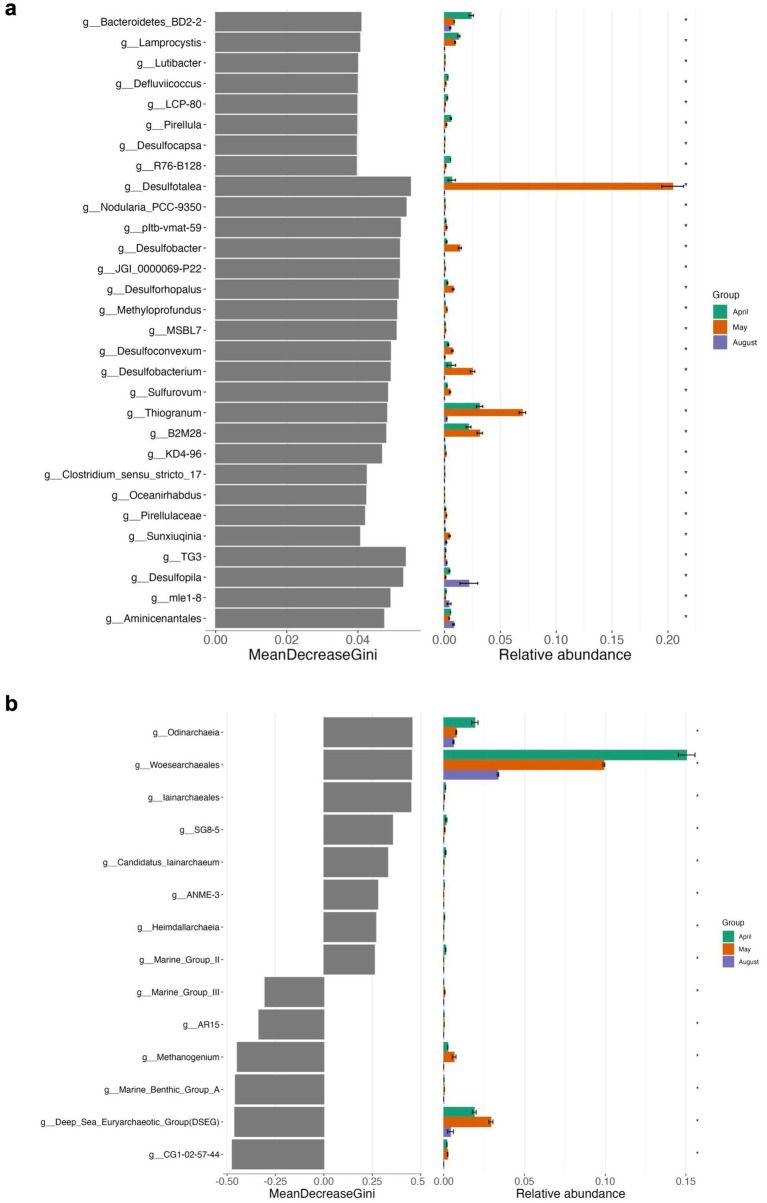
Differential abundance analyses using random forest testing on genus level for Bacteria **(a)** and Archaea **(b)** of top sediment incubations. The MeanDecreaseGini score represents a measure for the importance of a genus in the random forest model.

On a level of significance, taxa that were important for differentiating April, May and August incubations at the end of the experiment from each other were for April and May as opposed to August *Desulfobacterium*, *Thiogranum*, and B2M28, for May as opposed to the other experiments *Desulfotalea*, and for August *Desulfopila* (*Desulfocapsaceae*) (Figures 5, 7a, Supplementary Figure S2). The most important archaeal taxa distinguishing the April and May communities from the August top sediment slurries were *Woesearchaeales* and DSEG, while for May slurries with 2 to 5 cm sediments the most important taxon differentiating them from April and August incubations was the methanogen *Methanogenium* (*Methanomicrobiaceae*) (Figures 6, 7b).

**Figure 8:**
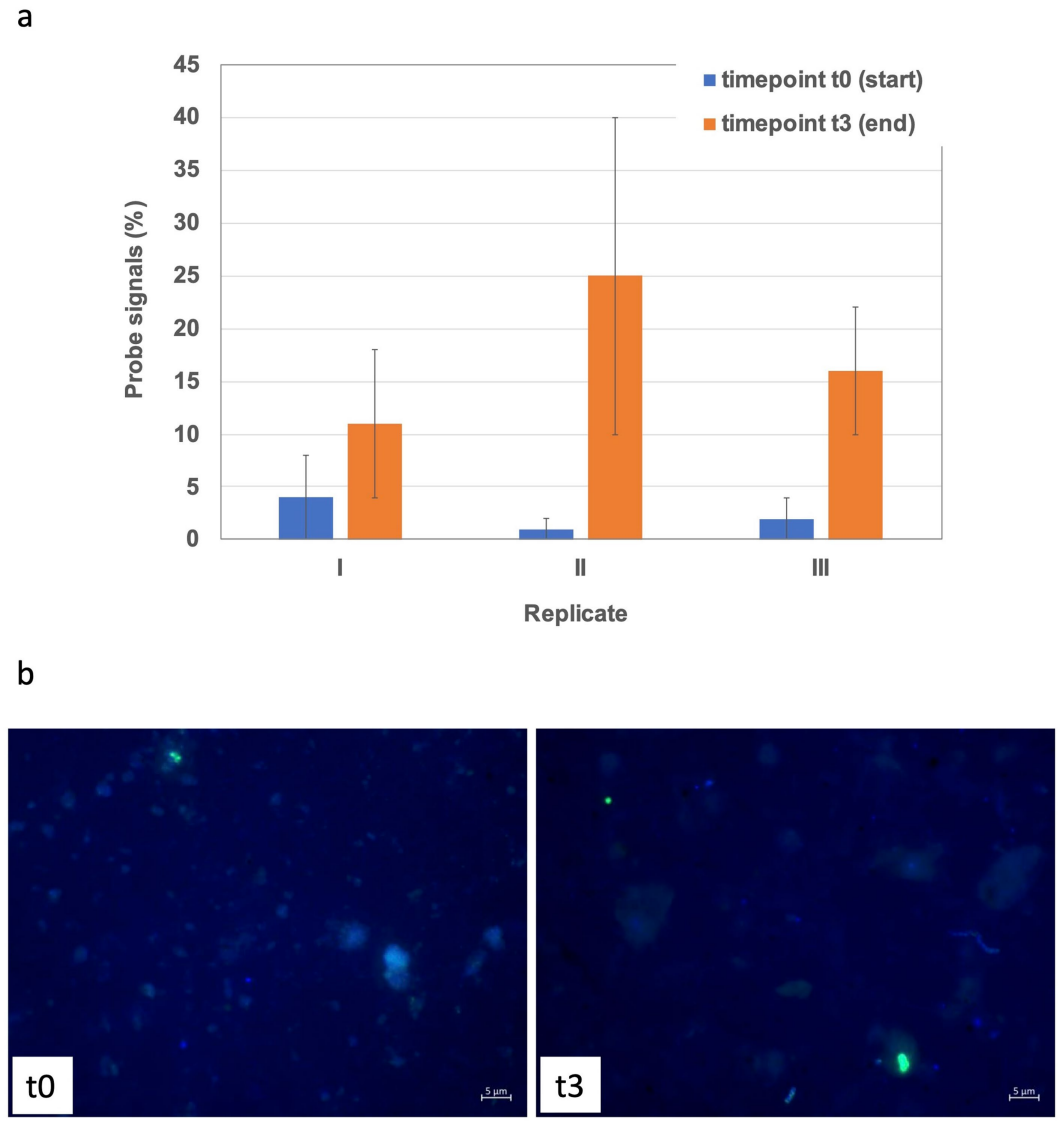
CARD-FISH based quantification of Archaea **(a)** in the May 18 to 22 cm slurry and exemplary microscopic CARD-FISH images **(b)** at the start and end of the experiments. Fluorescence signals are shown as overlays, where those of the archaeal probe ARCH915 are displayed in green, while DAPI signals representing all cells are shown in blue color.

*Bottom sediments*. April experiments exhibited enrichments, which primarily included differently strong shifts towards *Desulfobacteraceae* in all replicates and *Sulfurimonadaceae* in two out of the three replicates. For May experiments the trend between the three replicates was more consistent towards clade WCHB1-32 (*Prolixibacteraceae,* 14–17%), *Alkalibacteraceae* and *Desulfovibrionaceae* with variations in the abundance of the respective 16S rRNA gene amplicons of the latter two families between the three replicates. Replicate I was enriched towards 50% of *Alkalibacter* and 25% of *Halodesulfovibrio*, replicate II towards 29% (*Alkalibacter*) and 47% (*Desulfovibrio* 38% and *Halodesulfovibrio* 9%) and replicate III towards 40% (*Alkalibacter*) and 12% (*Halodesulfovibrio*), respectively (Figure 5, Supplementary Figure S2). Shifts in the August experiments were most consistent, where all replicates showed very consistent trends. In August bottom sediments no clearly dominating lineages were observed but rather numerous taxa became enriched such as SRB of *Desulfobacteraceae*, *Desulfosarcinaceae* and *Desulfocapsaceae*, organotrophic *Syntrophotaleaceae* and putative SOB of the *Thiotrichaceae* and *Sulfurimonadaceae* families (Figure 5). Overall, only minor changes were observed over time in the abundances of the different taxa forming the archaeal communities of the April and August bottom sediment incubations. In the May bottom sediment slurries however, a massive enrichment of 16S rRNA gene tags allocated to ANME-1b was striking - from <0.1% of all archaeal 16S amplicons at the start of the experiment to 24–35% at the end of the slurry experiments (Figure 6a). Simultaneously, quantitative CARD-FISH analyses revealed an increase of archaeal probe signals from <4% at the start of the experiments to up to 25% of all DAPI-stained cells at the end of the experiments (Figure 8). In detail, at the end of the enrichment experiment, May slurries with bottom sediments were mostly dominated by ANME-1a, ANME1-b, ANME-2a-2b (summing up to 27–38%), accompanied by a smaller proportion (6–8%) of 16S rRNA gene amplicons related to known traditional methanogens. The majority of the latter group was associated with unclassified members of the order *Methanofastidiosales* (6–8%). Other known methanogenic genera like *Methanosarcina* were identified at very low abundances (<0.2%, each) in the bottom sediment incubations in May (Figure 6). In all other bottom sediment slurries ANME remained below 0.03% of archaeal 16S rRNA amplicons, while abundances of methanogens (again predominantly *Methanofastidiosales*) in August bottom sediment incubations exceeded those of May with 12 to 13% in total (Figure 6a). A closer look at only the “traditional” methanogens and ANME distribution of 16S rRNA gene amplicons demonstrated that the abundance of ANME-1b decreased almost completely in April bottom sediments during incubations (Figure 6b). The fact that the April bottom sediment incubations were the only ones exhibiting no noticeable increase in 16S rRNA gene amplicons of methanogens, could indicate that methanogenesis was negatively affected by increased H_2_ concentrations and/or the overall redox state in the incubations. Despite the high proportions and enrichment of methanogens in the August bottom sediment slurries (Figure 6a), the abundance of ANME ceased completely in two of the three replicates (Figures 6a,b), indicating that the presence of substrates was not decisive of AOM activity and growth of ANME in our sediment incubations.

**Figure 9:**
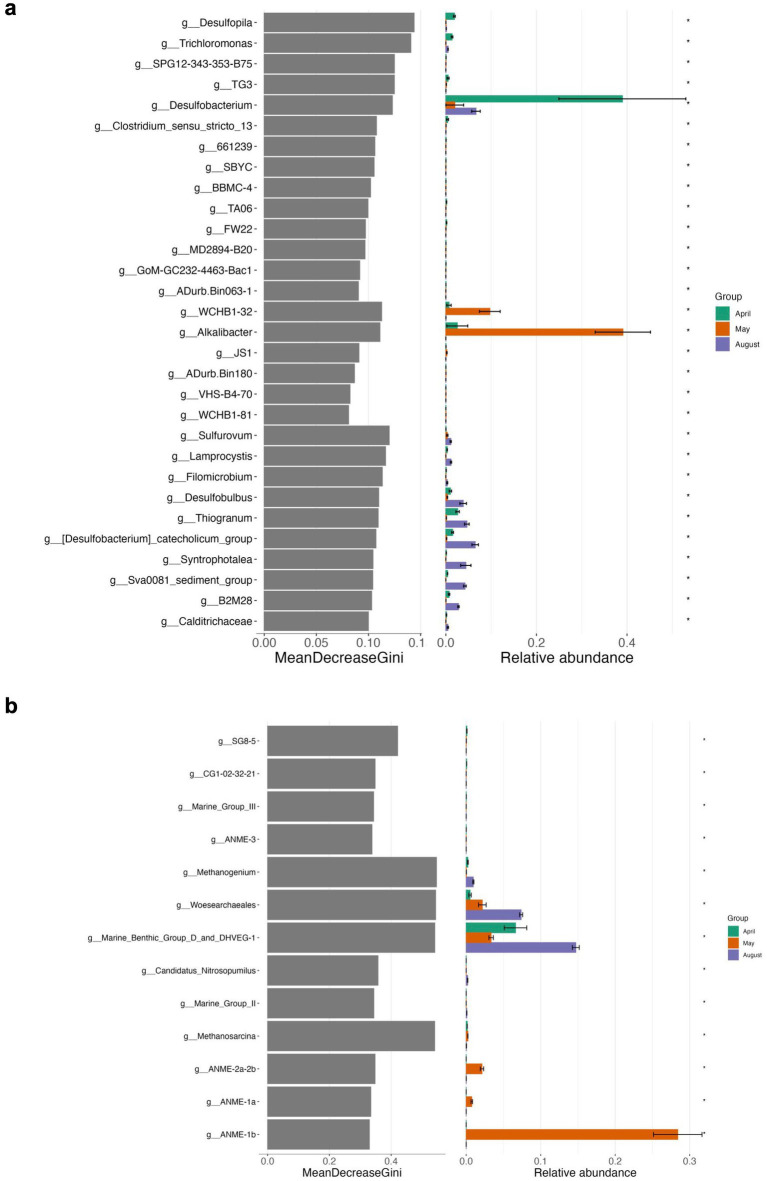
Differential abundance analyses using random forest testing on genus level for Bacteria **(a)** and Archaea **(b)** of bottom sediment incubations. The MeanDecreaseGini score represents a measure for the importance of a genus in the random forest model.

In summary, bottom sediments were mostly affected by bacterial rather than archaeal community shifts (exception May) where the most important groups differentiating the slurry experiments from each other at the end of the experiments were *Desulfobacterium* (*Desulfobacteraceae*) (April), WCHB1-32 (*Prolixibacteraceae, Bacteroidales*) *Desulfovibronaceae* and *Alkalibacter* (*Alkalibacteraceae, Firmicutes*) (May) and phylogenetically diverse SRB such as Sva0081 (*Desulfosarcinaceae*), *Desulfobacterium catecholicum* (*Desulfocapsaceae*), *Desulfobulbus* (*Desulfoblbaceae*) and *Syntrophotalea* (*Syntrophotaleaceae*) as well as potential SOB such as *Thiogranum* (*Ectothiorhodospiraceae*), *Thiotrichaceae,* B2M28 and *Lamprocystis* (*Chromatiaceae*) (August) (Figures 5, 9a, Supplementary Figure S2). In contrast, among the Archaea, on a level of significance only August slurries demonstrated enrichments in Marine Benthic Group D, DSEG, *Woesearchaeales* and some *Methanogenium* and May bottom sediments enrichments in ANME-1b during incubations (for details see also above, Figures 6, 9b).

**Figure 10:**
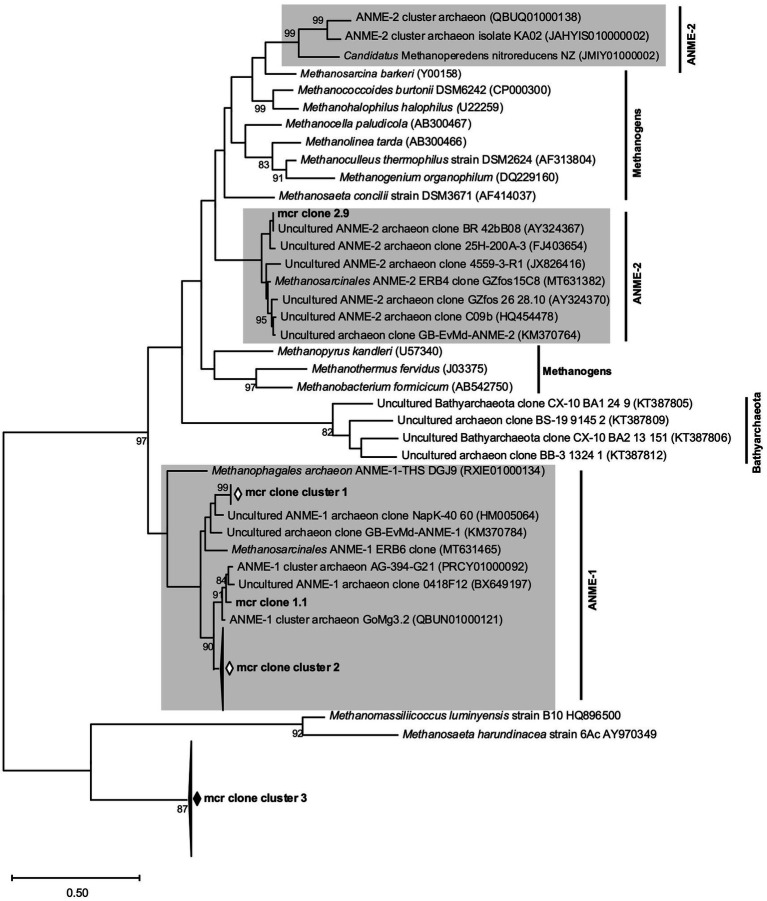
Phylogenetic tree of *mcr* genes from May bottom sediment incubations generated using ANME specific primer pairs (mcr clones) as well as reference mcr genes from methanogens, ANME-1, ANME-2 and Bathyarchaeota. The scale bar denotes the number of substitutions per nucleotide position and bootstrap values are only indicated if greater than 80%.

To better understand and characterize the conspicuous enrichment of ANME genes encoding Mcr (methyl coenzyme M reductase) were amplified from May slurries with 18 to 22 cm sediments. Generic primers used for detecting *mcr* genes from classical euryarchaeotal methanogens did not yield any products, neither at the start nor at the end of the experiments, albeit the positive control *Methanosarcina mazei* gave signals (data not shown). ANME-1 and ANME-2 specific *mcr* primers gave significant PCR products at the end of the experiments but gave no products at the start of the experiment, further confirming the enrichment of ANME during incubation at increased H_2_ concentrations. Phylogenetic analyses of sequenced *mcr*-gene clones documented three distinct clone clusters (all generated with ANME-1 specific primers) placed within the ANME-1 *mcr* gene cluster (Figure 10). Clones generated with ANME-2 specific primers were less diverse: only one of them clustered together with other ANME-2 *mcr* genes, while the rest (18 clones) represented one large cluster exhibiting highest similarity with the *mcr* genes of the methanogenic *Methanosaeta harundinacea* and *Methanomassillicoccus luminyensis* species (Figure 10).

## Discussion

### Linking hydrogen consumption rates with shifts in the microbial community compositions

Our incubation experiments revealed the capability of microbial organisms and communities to adapt to increased H_2_ concentrations. The shifts in microbial diversity were largely consistent in triplicate experiments. However, we observed large phylogenetic differences in the initial and incubated samples depending on the time of sediment sampling (April, May or August), indicating a marked seasonality in line with previous observations (Perner et al., 2022). We predominantly observed a season-dependent enrichment of organisms, which became dominant during incubations, although they were below detection limit at the start of the experiment. Furthermore, during most slurry incubation experiments microbial diversity - as shown by Shannon diversity indices - decreased, with the most pronounced effects notable in the May bottom sediments (Supplementary Figure S4). This observation may not only result from the elevated H_2_ concentrations but also from the “bottle effect,” frequently observed in lab-based incubation experiments that are cut off from the *in situ* supply of OM, nutrients, electron donors and acceptors and leading to significant shifts of microbial communities (e.g., Baltar et al., 2012; Ionescu et al., 2015). Furthermore, the artificial seawater medium used for setting up the sediment slurries differs from the geochemical composition of the respective pore waters of the incubated sediment layers (e.g., in hydrogen sulfide concentrations), most likely also influencing microbial activity and communities. Overall, this indicates that incubation at the offered H_2_ concentrations and medium conditions does not reflect *in situ* H_2_ availability and consumption, but rather provides insights into the H_2_ oxidizing potential of the respective microbial communities. It should be noted, though, that these experiments currently represent the closest quantitative approximation to *in situ* H_2_ consumption rates, as *in situ* measurements are hampered by the commonly low H_2_ concentrations and continuous microbial H_2_ production in anoxic sediments.

### Enrichment of various SRB was obvious in most incubations

While the incubations were carried out with sediment samples obtained under different in situ redox conditions due to seasonally occurring bottom water hypoxia and shifts in biogeochemical gradients in near-surface sediments, redox conditions in the incubations were essentially targeted to enable microbial SR (initial SO_4_^2−^ concentration ~ 15 mM). *Desulfatiglandaceae* were most abundant among putative sulfate-reducing microorganisms (SRM) in situ and present in all initial slurries and at both sampling depths, but their relative abundance decreased strongly during incubations at increased H_2_ concentrations. *Desulfatiglandaceae* are considered as highly versatile microorganisms which can metabolize a large variety of organic substrates and also might be able to adapt to non-SR metabolisms (acetogenesis, fermentation) in the absence of sulfate (Jochum et al., 2018). At the end of the H_2_ amended incubations however, other putative SRMs were enriched with strong dependence on seasonality. Members of the respective genera, such as *Desulfobacterium*, *Desulfotalea* and *Desulfovibrio* are commonly described as metabolically versatile SRB (including autotrophic lifestyles) with the ability to use H_2_ as an energy source (Brysch et al., 1987; Knoblauch et al., 1999; Baffert et al., 2019). The enrichment of initially low abundant SRM and the (compared to initial sediment communities) decreased abundances of Desulfatiglandaceae in the H_2_-spiked incubations may indicate that H_2_ is not a major electron donor for SRM in Boknis Eck sediments in the sampled seasons.

### The response to H_2_ addition is season-dependent

The top sediments of April and May exhibited a similar shift in microbial community composition among the classifiable bacterial families (Figure 5). Yet, May slurries with top sediments consumed around twice as much H_2_ per cell (Figure 3b) during the same incubation period than the April slurries kept under the same conditions. This may be explained by a microbial community adapted to a higher H_2_-availability in the sediments. Phytoplankton blooms are known to occur in late winter/early spring at Boknis Eck (Smetacek et al., 1984; Bange et al., 2011) and synthesizing fresh OM, which essentially rain down on the seafloor. Input of labile OM and rising temperatures from spring onwards stimulate increased OM degradation, thereby producing H_2_ and consuming primarily O_2_, consequently, giving rise to microbes catalyzing hydrogenotrophic processes. For example, H_2_ consumption in the May top sediment slurries appeared to be related to members of the *Desulfotalea* genus (*Desulfocapsaceae*), which are known H_2_ oxidizers (Knoblauch et al., 1999) and made up 20% of the bacterial 16S rRNA gene tags in May but were absent in two of the three replicates in April top sediment incubations (Supplementary Figure S2). Within the *Desulfocapsaceae* family members of the facultatively H_2_ oxidizing [Desulfobacterium] catecholicum group (type strain reclassified as *Desulfocastanea* within the same family) (Galushko and Kuever, 2019) showed the highest enrichment (up to 12% on the genus level) in April top sediment incubations. Thus, H_2_ consumption in the incubation experiments was essentially coupled to SR, which is in accordance with sulfate availability and the relative dominance of microbial SR in top sediments compared to other anaerobic processes. However, albeit only in small relative abundance, the May top sediments became also enriched in methanogenic *Methanogenium* and DSEG as important taxa for differentiating the slurry community from the other top sediment incubations (Figures 6, 7b). Consequently, besides SRM, also other organisms are directly or indirectly affected by increased H_2_ supply and likely consume H_2_, metabolic intermediates or end-products (e.g., acetate or methane). Additionally, the top April and May sediment incubations were characterized by approximately 30 and 15%, respectively, of low-abundant and/or unclassified Bacteria (Figure 5b). This uncalled-for group likely masks H_2_ utilizing ability which remains, with our current tools, undetectable. As expected in the light of substrate competition with SRM, in all sediment incubations hydrogenotrophic methanogens were (at most) only present in small numbers. Instead, uncultured members of the methylotrophic *Methanofastidiosales* (Nobu et al., 2016) dominated within the methanogenic communities (Figure 6b). Despite differing abundances of methanogens already at the start of the experiments, April and May top sediment slurries demonstrated comparable methane contents in the first 2 weeks of incubation (Figure 4), which may be related to the higher cell counts in the April top sediments. The ongoing increase of methane concentrations during the second half of the May top sediment incubations may be explained by the initial starting conditions providing labile OM input, lower O_2_ concentrations in the bottom waters and higher temperatures compared to the April incubations (see above).

In total, the microbes in the May bottom sediment slurries consumed 50% more H_2_ than those from April sediments, but compared to May top sediments H_2_ consumption was roughly halved (Figure 2, Supplementary Figure S1). Per volume H_2_ consumption in April bottom sediments was less than 50% of that from April top sediment incubations (Figure 3a). However, when considering the per cell rate, the difference in H_2_ consumption between the top and bottom sediment incubations of both April and May were less pronounced (Figure 3b). The lower H_2_ consumption rates in April bottom slurries coincide with enrichments of putatively autotrophic *Sulfurimonas* (55% in replicate II and 15% in replicate III) and *Desulfobacterium* spp. (40% I replicate I, 15% in replicate II and 65% in replicate III) (Supplementary Figure S3a). *Sulfurimonas* spp. are hallmarked by versatile metabolisms and are capable of oxidizing H_2_, hydrogen sulfide, elemental sulfur, thiosulfate, sulfite coupled to reduction of nitrate, nitrite, manganese oxide or O_2_ and can grow autotrophically or with acetate or various organic compounds (Han and Perner, 2014, 2015; Han and Perner, 2016; Henkel et al., 2021). Physiological experiments demonstrate that *Sulfurimonas* consumes H_2_, but also that significant growth requires a sulfur source. If offered, it prefers reduced sulfur species as energy source, coupling their oxidation to nitrate reduction (Han and Perner, 2014). *Desulfobacterium* spp. can couple sulfate and thiosulfate respiration with lactate, H_2_ oxidation with SR or perform pyruvate fermentation (Neretin et al., 2003). Members of this group have also been associated with dimethylsulfoxide (DMSO) reduction (Jonkers et al., 1996). Although our experiments do not resolve if and which electron acceptors other than sulfate became available in the course of the incubations, the dominance of *Sulfurimonas* and *Desulfobacterium* spp. in bottom April slurries suggests active sulfur cycling between the taxa when starting sediments are exposed to anoxic conditions.

Increased per cell H_2_ consumption rates of May bottom sediments align with shifts towards SRB of the *Desulfovibrionaceae* (*Desulfovibrio* and *Halodesulfovibrio*), *Prolixibacteraceae* (WCHB1-32) and *Alkalibacteraceae* (*Alkalibacter*)—at the start they made up less than 1% and at the end between 12 and 47%, ca 6 to 14% and 28 to 50%, respectively, of all bacterial 16S rRNA gene amplicons (Figure 5a). This is a notable difference between the communities from May bottom sediments and the April slurries and as such to all other slurries. *Alkalibacter* spp. are not known to utilize H_2_ or sulfate but instead ferment distinct organic compounds (e.g., mono- and disaccharides released in the course of polymer degradation), producing formate, acetate, ethanol, hydrogen and CO_2_ and favoring alkaline environments (Garnova et al., 2004; Namirimu et al., 2022). In contrast, *Desulfovibrio* can couple oxidation of lactate, formate and H_2_ to SR (Postgate and Campbell, 1966). Some strains can grow autotrophically (Sánchez-Andrea et al., 2020) or appear to be involved in DMSO reduction (Jonkers et al., 1996). Some strains are also involved in iron reduction (Gonzalez et al., 2024) which could explain why sulfide concentrations experience a low in the starter sediments between 18 and 22 cm (cf. Figure 1c). Strains have also been documented to be involved in replacing anhydrite (Ca[SO_4_]) with authigenic carbonates (Zueblin, 1988). When either OM or methane (in conjunction with ANME) is available, bicarbonate is produced, increasing alkalinity and causing an oversaturation of calcium- and carbonate-ions; thus, forming secondary carbonate precipitates (Rouwendaal et al., 2023). If this were the case then the enrichment of *Alkalinibacteraceae* could be explained.

Transient amounts of methane, although low compared to H_2_ supply and consumption were slightly higher compared to other experiments. In May bottom sediments, methane reached a maximum after 1 week of incubations and was then consumed rapidly but started accumulating again slowly towards the end of the experiment. Although no methanogenic *mcr* genes were identified in the respective sediment slurries, uncultured members of the methylotrophic *Methanofastidiosales* (Nobu et al., 2016) made up ~6–8% of the archaeal community, similar to what was found in most other slurries (Figure 6a). It remains unclear though, why in this specific slurry methane production was slightly higher than in the other respective slurries. A possible explanation may be the presence of alternative “non-traditional” methanogens: In the last years evidence has grown that methane metabolisms might be more widespread among Archaea than previously assumed. For example, homologues of genes encoding enzymes involved in hydrogenotrophic and methylotrophic methanogenesis have been detected in Bathyarchaeota and Verstraetarchaeota (Evans et al., 2015; Vanwonterghem et al., 2016; Zhou et al., 2018). Previous experiments conducted with Boknis Eck sediments recognized that under the tested conditions methylated substrates or methanol rather than H_2_ served as base for methanogenesis, yet phylogenetic profiling was not conducted and thus the archaeal lineages responsible for the methanogenic activity were not identified (Maltby et al., 2018). Bathyarchaeota were present in all our incubated sediment slurries, but no specific enrichment could be noted for the May 18 to 22 cm sediments (Figure 6a). However, different bathyarchaeotal populations may be involved in producing methane during specific slurry conditions. Then again, this could be visualized by transcriptomic data, which are lacking for the sediment slurries set up in this study. On the other hand, previous work has proposed that ANME may be capable of methanogenesis under specific environmental conditions (Biddle et al., 2012; Kevorkian et al., 2021), as they possess the enzymatic repertoire for hydrogenotrophic methanogenesis but reverse it to perform AOM (Timmers et al., 2017). Since this reverse methanogenesis needs to be highly exergonic and may rely on inter-species H_2_ transfer, (just like fermentation) it is not feasible under high H_2_ concentrations (Hoehler et al., 1994; Coon et al., 2023), which may be the cause for the decrease of ANME-abundances in August bottom sediments. Furthermore, analyses of various metagenome-derived genomes revealed the presence of [NiFe]-hydrogenases in ANME-1a, ANME-1b and ANME-1c clades, but it still remains unclear, whether these typical H_2_ uptake enzymes are metabolically involved or just a hereditary remnant in ANME-1 (Benito Merino et al., 2022; Laso-Pérez et al., 2023).

Given the massive enrichment of ANME in the May bottom incubations within only 3 weeks and the elevated methane concentrations over a large part of the incubation period, it is tempting to speculate that at least parts of the ANME populations may be indeed operating hydrogenotrophic methanogenesis and that methanogenic microorganisms can effectively compete for hydrogen under sulfate reducing conditions. However, this would be highly uncommon and conspicuous, since methanogenesis is thermodynamically unfavorable relative to sulfate reduction and, thus, usually limited to turnover of non-competitive methanogenic substrates (e.g., methylamines) in natural habitats (e.g., Oremland and Polcin, 1982). Further, we note, that methane concentrations remained low compared to consumed H_2_ throughout the experiments with only small changes compared to control incubations. Thus, if enrichment of ANME is related to hydrogenotrophic methanogenesis, it needs to be balanced by methane consumption through AOM. This would involve some very efficient niche partitioning of forward and backward catalyzing organisms and requires further investigation. Still, while cryptic methane cycling under sulfate-reducing conditions was reported in the past (Krause et al., 2023), it is, again, usually related to methanogenesis from non-competitive substrates and only of minor importance for net substrate turnover at non-limiting sulfate concentrations (Kevorkian et al., 2022). Methane production from non-competitive substrates such as methanol has been observed in ANME-1 dominated microbial mats (Bertram et al., 2013) and may also be a putative metabolic pathway for ANME in our sediment incubations. However, given the presence of methylotrophic methanogens in all sediment slurries, methylotrophic methanogenesis of ANME – with or without cryptic methane cycling – may not explain why the ANME enrichment is limited to incubations with May bottom sediments. Taking into account that in the past, methanogenic growth of ANME was mainly inferred from indirect evidence such as *mcrA* gene copy numbers in methanogenic sediments below the sulfate–methane transition zone (SMTZ) (e.g., Lever et al., 2023; Deng et al., 2025), and that there is no direct evidence of hydrogen-based methanogenic growth of ANME-1 from incubation studies (e.g., Benito Merino et al., 2022), it needs to be considered that apparent hydrogenotrophic growth of ANME is potentially unrelated to methanogenesis, but instead either to the turnover of metabolic intermediates or products from dissimilatory sulfate reduction (e.g., S(0), Fang et al., 2020; Wang et al., 2023) or reactive compounds being present and characteristic in May sediments due to the beginning decrease of oxygen concentrations in overlying bottom waters. Alongside the dramatic increase in ANME, members of *Desulfovibrionaceae* and *Alkalibacter* became considerably enriched in the course of the May bottom incubations. Previous experiments have suggested that sulfate reducing H_2_ oxidizers and hydrogenotrophic methanogens induce an increase in pH related to intense proton- and bicarbonate utilization (Dopffel et al., 2023). Hence, we posit that here *Desulfovibrio* potentially interacting with a methanogen from the *Methanofastidiosales* or Bathyarchaeota populations produce alkalinity that favors *Alkalibacter*. Interestingly, despite similar shifts in *Desulfovibrionaceae* abundances in August top sediment incubations, May bottom slurries are the only slurries where the explicitly high enrichment of *Alkalibacteraceae* is featured, although alkalinity production through hydrogenotrophic SR and methanogenesis is expected to be largely similar in other incubations with intense H_2_ consumption.

Overall, the distinct enrichment of both ANME and *Alkalibacter* in May bottom sediments are considered as indicative of specific properties of starter sediments. These specific properties of May bottom sediments may be related to the shift from oxic to anoxic conditions in the overlying bottom waters compared to April experiments. This change could induce community shifts and the availability of additional electron acceptors. It could be further related to abiotically or metabolically produced compounds or viruses modulating microbial activity, as it has been documented that certain viruses appear specific to, e.g., ANME (Laso-Pérez et al., 2023).

The August sediment slurries consumed the most H_2_ during incubations (Figures 2, 3). The August top sediment incubations were characterized by a quite different bacterial composition relative to the slurries from April and May. Apart from the enrichment in *Desulfovibrionaceae* and *Alkalibacteraceae*, a shifting towards *Desulfopila* and non-SR organotrophs was observed. *Desulfopila* spp. use a broad range of organic electron donors for SR, are involved in iron cycling and can also grow with H_2_ chemolithoautotrophically (Gittel et al., 2010; Beese-Vasbender et al., 2015).

The August bottom sediments with the overall highest H_2_ consumption per cell were the most phylogenetically diverse assemblage containing SRB and SOB as well as shifts towards *Methanogenium* and MBG-D. It may be this higher phylogenetic diversity that facilitates the interactions and thus metabolic exchange furthering elevated H_2_ consumption. Moreso, several putative SOB encode hydrogenase genes on their genome (e.g., Anantharaman et al., 2013; Tengölics et al., 2014), which may partake in consuming the H_2_. The reason for this distinct development of the community in August sediments may be related to more established reduced conditions in sediments as opposed to April and May sediments. Possible reasons could include the availability of compounds released from mineral grains, such as phosphate and metals like iron or manganese.

## Conclusion

Although all of the incubated sediments originate from the same location, the seasonal variations in sedimentary community composition and pore water geochemistry set distinct starting conditions for the H_2_ enrichments. Sediments exposed to hypoxic bottom water conditions favor a microbial starter community with the highest H_2_ oxidation potential. This may be related to the overall redox state of the sediment and interstitial waters and more specifically to interactions with dissolved and solid sulfur and iron species. The incubation experiments indicate the breadth of biotic and abiotic interactions that are taking place under anoxic conditions, where minerals and likely OM content and quality affect metabolisms, microbial activity and thus community compositions. Even though most of the observed H_2_ oxidation potential can be related to hydrogenotrophic SRB, the putative involvement of massively enriched ANME in the H_2_ cycling of May bottom sediment incubations is conspicuous. The tools and methods applied in this study could not complete the picture of the complex microbial and especially metabolic networks involved in our observations. Detailed metagenomic and/or metatranscriptomic analyses could contribute indications of putatively active metabolic processes but also cannot provide a clear proof of the involvement. Thus, further ecophysiological experiments (extending the here described experiments with, e.g., quantification of hydrogen sulfide production and stable carbon isotope analyses of methylated substrates and methane) are needed to obtain a comprehensive picture of the microbial processes involved in hydrogen and methane cycling taking place in sediments underlying seasonally hypoxic bottom waters.

## Data Availability

The datasets presented in this study can be found in online repositories. The names of the repository/repositories and accession number(s) can be found at: https://www.ncbi.nlm.nih.gov/, PRJNA1213115.
