# Supplementary material for: Microbial hydrogen oxidation potential in seasonally hypoxic Baltic Sea sediments
Source: Front Microbiol. 2025 Apr 4;16:1565157. doi: 10.3389/fmicb.2025.1565157 (PMC12007115; doi:10.3389/fmicb.2025.1565157)

**Supplementary information for:**

**Microbial hydrogen oxidation potential in seasonally hypoxic Baltic Sea sediments**

Adam-Beyer, Nicole ^1^, Deusner, C.^2^, Schmidt, M^2^ and Mirjam Perner^1*^

^1^Geomicrobiology, Marine Geosystems, GEOMAR Helmholtz Centre for Ocean Research Kiel, Wischhofstr. 1-3, 24148 Kiel, Germany

^2^Benthic Biogeochemistry, Marine Geosystems, GEOMAR Helmholtz Centre for Ocean Research Kiel, Wischhofstr. 1-3, 24148 Kiel, Germany

Key words: hydrogen oxidation, hydrogen consumption, marine sediments, Methane metabolism, methanogenesis, AOM, ANME, SRB

This file contains: Supplementary figures S1-S4

**Supplementary Figure S1**. Monitored hydrogen concentrations in head spaces of each individual sediment slurry.

**
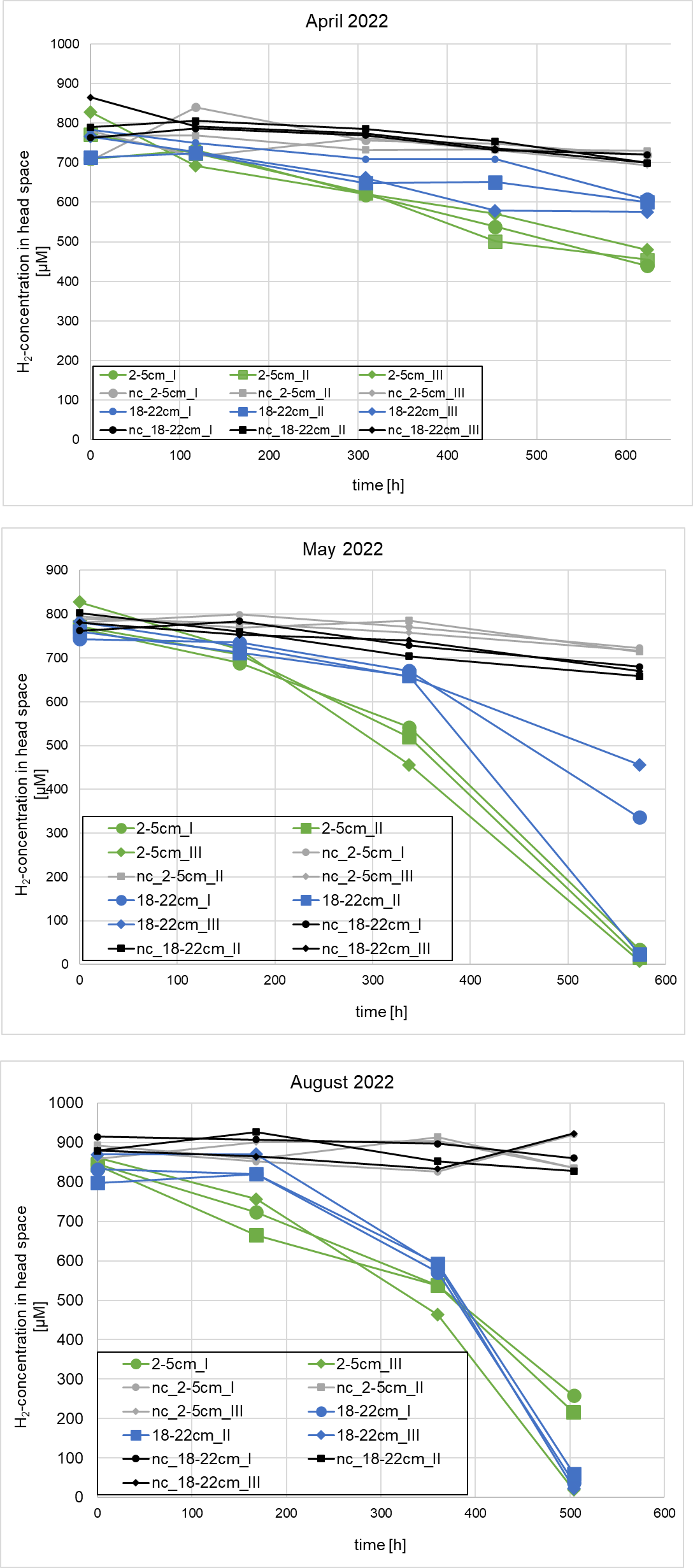
**

**Supplementary Figure S2.** Taxonomy plots based on RNA of 16S amplicons on a genus level for 30 most abundant bacterial taxa (a) and putative Sulfate Reducing Bacteria (SRB) (b).


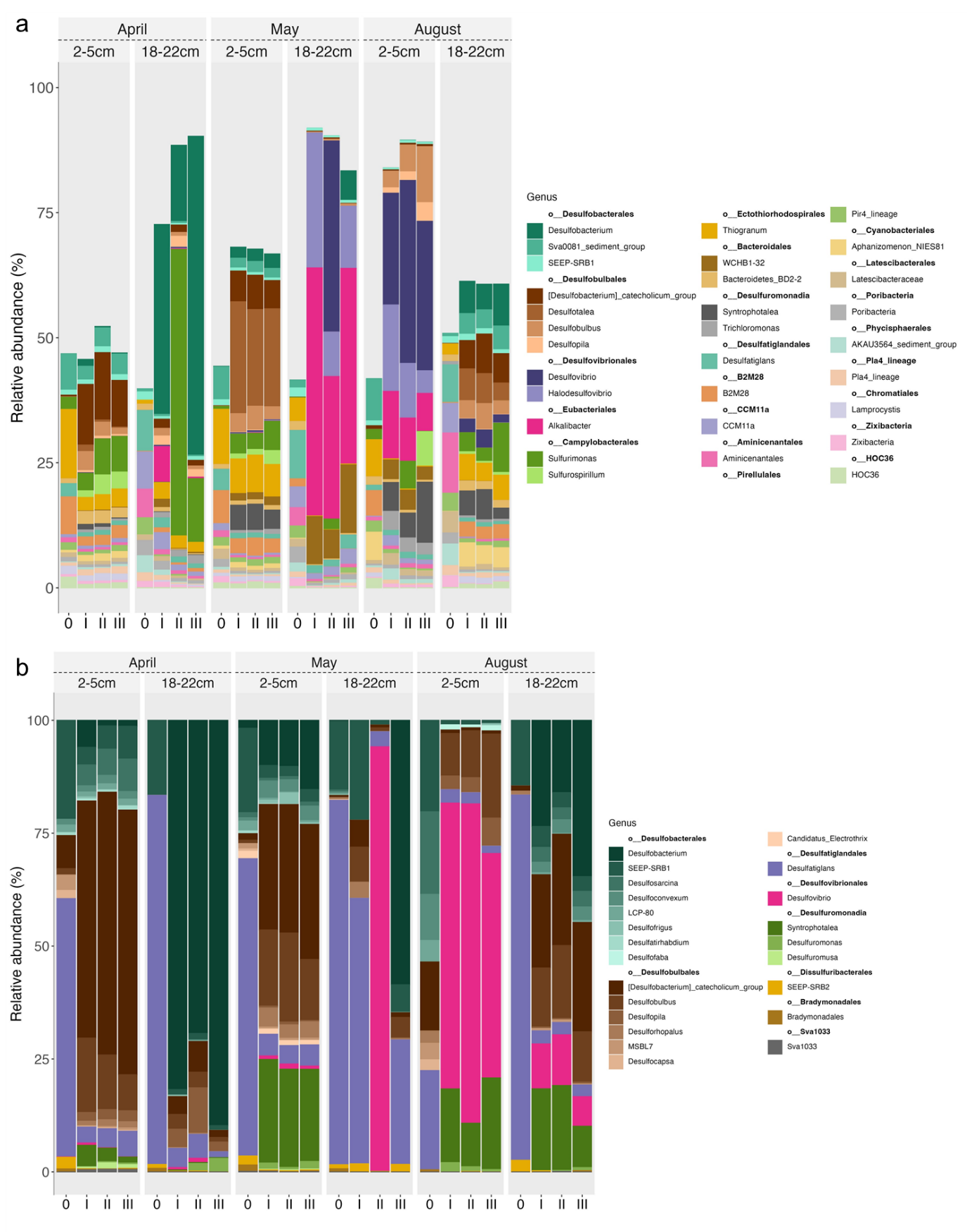


The number at the bottom of the graph denotes community at the start of the experiments (0) and at the end of the experiment in triplicates (I, II, III). The subplot of SRB (b) is scaled to 100%.

**Supplementary Figure S3.** Cell counts at the start (t_0_) and end (t_end_) of the sediment slurry incubations.


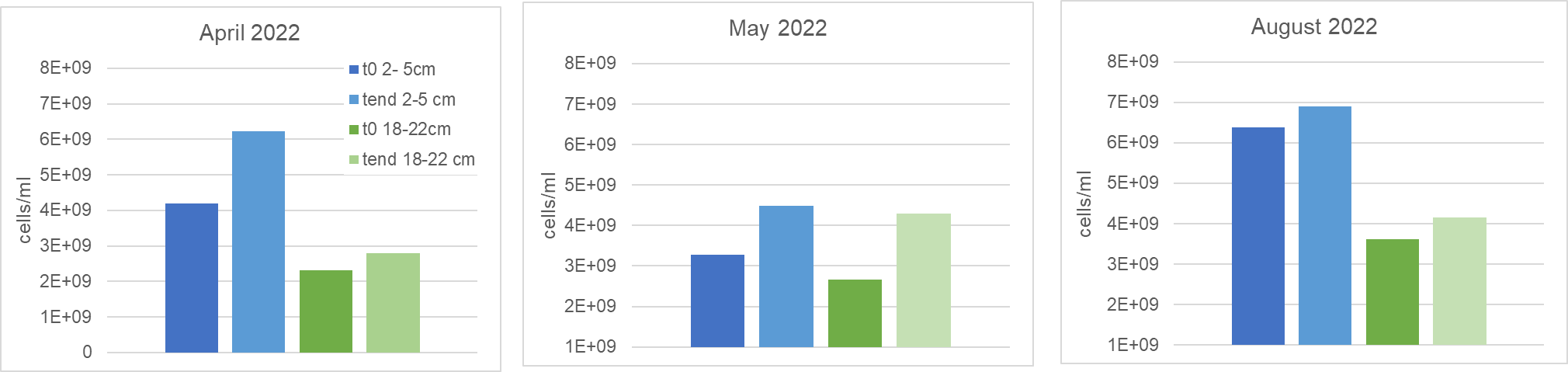


**Supplementary Figure S4.** Shannon Diversity Index of 16S tags from bacterial (a) and archaeal (b) communities.


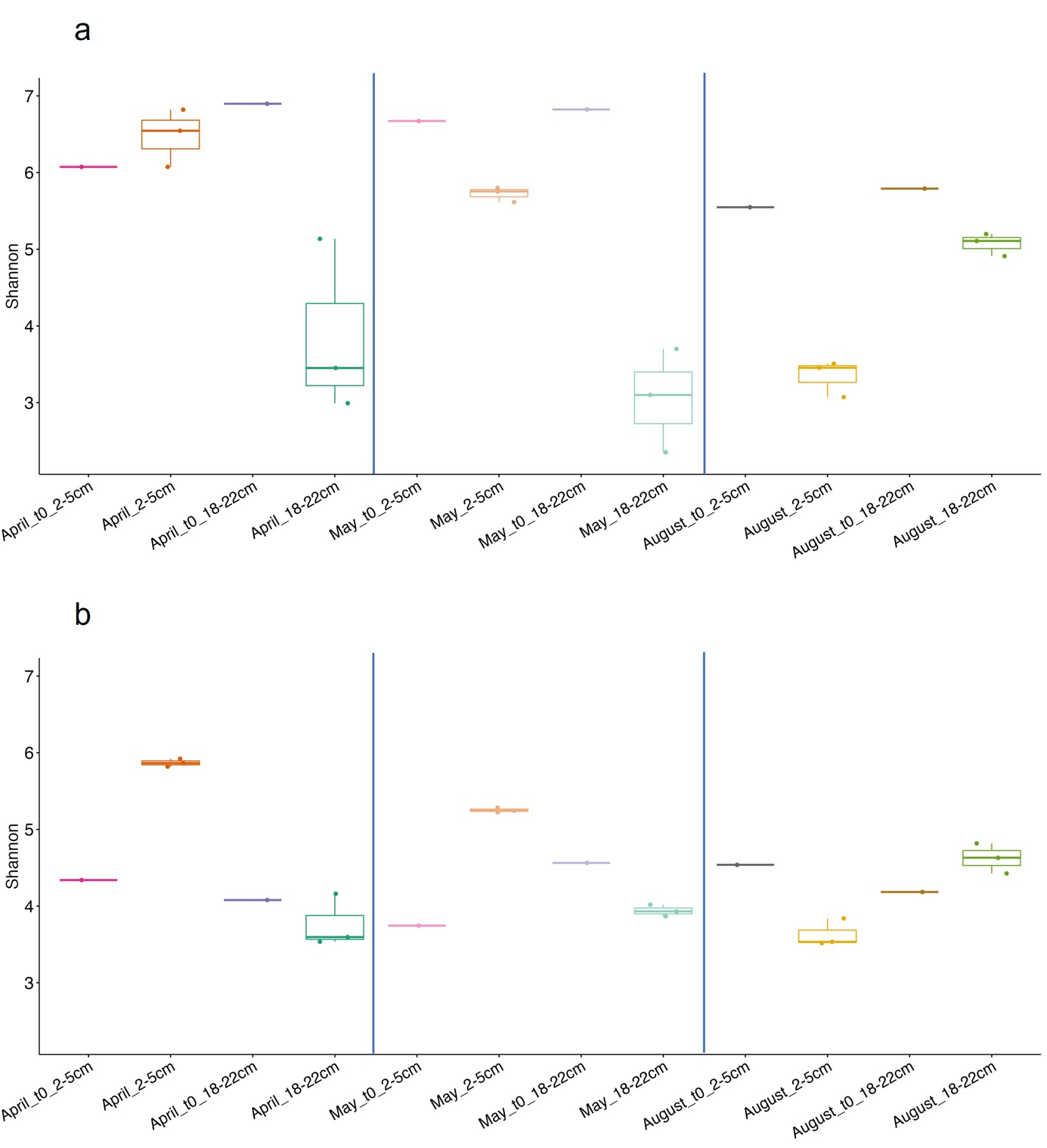

Supplement: Supplementary file 1 [file Supplementary_file_1.docx]
